# Investigation of Hydration States of Ionic Liquids by Fourier Transform Infrared Absorption Spectroscopy: Relevance to Stabilization of Protein Molecules

**DOI:** 10.1021/acs.langmuir.2c02851

**Published:** 2023-02-08

**Authors:** Navin Rajapriya Inbaraj, Subin Song, Ryongsok Chang, Kyoko Fujita, Tomohiro Hayashi

**Affiliations:** † Department of Materials Science and Engineering, School of Materials Science and Chemical Technology, Tokyo Institute of Technology, 4259 Nagatsuta-cho, Midori-ku, Yokohama-shi, Kanagawa-ken 226-8502, Japan; ‡ Department of Pathophysiology, Tokyo University of Pharmacy and Life Sciences, 1432-1 Horinouchi, Hachioji, Tokyo 192-0392, Japan

## Abstract

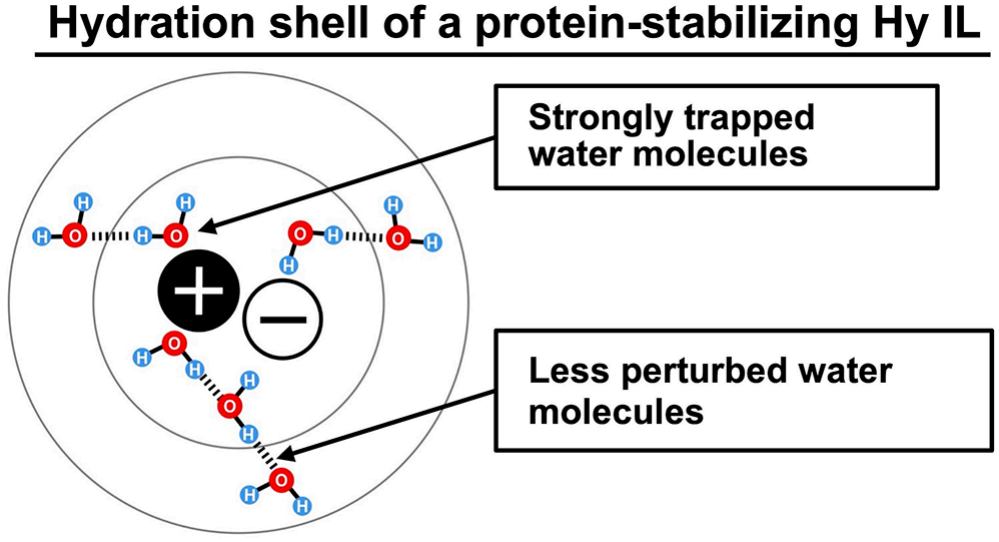

Among many kinds of ionic liquids, some hydrated ionic liquids (Hy ILs) have shown an exceptional capability to stabilize protein molecules and maintain their structure and functions over a long period. However, the complex IL–water interaction among these protein-stabilizing Hy ILs has yet to be elucidated clearly. In this work, we investigate the origin of the compatibility of ionic liquid with proteins from the viewpoint of hydration structure. We systematically analyzed the hydrogen-bonding state of water molecules around ionic liquid using Fourier transform infrared absorption (FT-IR) spectroscopy. We found that the native hydrogen-bonding network of water remained relatively unperturbed in the protein-stabilizing ILs. We also observed that the protein-stabilizing ILs have a strong electric field interaction with the surrounding water molecules and this water–IL interaction did not disrupt the water–water hydrogen-bonding interaction. On the other hand, protein-denaturing ILs perturb the hydrogen-bonding network of the water molecules to a greater extent. Furthermore, the protein-denaturing ILs were found to have a weak electric field effect on the water molecules. We speculate that the direct hydrogen bonding of the ILs with water molecules and the strong electric field of the ions lasting several hydration shells while maintaining the relatively unperturbed hydrogen-bonding network of the water molecules play an essential role in protein stabilization.

## 1 Introduction

Ionic liquids (ILs) are molten salts composed purely of ions and are generally considered to have a melting temperature below 100 °C.^1^ The ions are large and asymmetrical, which prevents the dense packing to achieve a crystalline phase at relatively lower temperatures. Some ILs remain liquid even at ambient temperature and are called room-temperature ILs.^2^ Due to the interionic interaction and low melting temperature, ILs have unique physicochemical properties: negligible vapor pressure,^3^ thermal stability up to 400 °C,^4^ wide liquidus range (>200 °C),^5^ and superior ionic conductivity.^6^ The physicochemical properties of IL enable a wide range of applications as an electrolyte,^7^ lubricant,^8^ thermal fluid,^9^ separating agent,^10^ etc.

Recently, several IL and water mixtures were investigated for their protein-stabilizing capability. For example, choline dihydrogen phosphate [Ch][dHp] mixed with 20 wt % water stabilized lysozyme molecules for up to a month with higher thermal stability and only 20% loss of activity.^11^ The same IL–water mixture stabilized cytochrome *c* for up to 1 year without affecting its native structure.^12^ Interestingly, several protein-stabilizing IL–water mixtures showed a unique phase transition behavior, cold crystallization (CC), during the slow heating of the supercooled amorphous phase.^13^ This CC behavior of IL–water mixtures could be detected in differential scanning calorimetry (DSC) as an exothermic peak in the thermogram. For example, [Ch][dHp], a protein-stabilizing IL mixed with water at a molar ratio of 7:1, water to IL, showed a CC behavior at −80 °C. However, [Ch][dBp], a protein denaturant, did not show CC behavior, irrespective of the water content.^13^ The authors also suggested that CC could be used as a screening method to determine the protein-stabilizing capability of Hy ILs. Similarly, CC behavior was also observed in biocompatible polymer–water systems of poly(ethylene glycol),^14^ poly(2-methoxyethyl acrylate),^15,16^ poly(tetrahydrofurfuryl acrylate),^17^ and poly(2-methacryloyloxyethyl phosphorylcholine).^18^ The authors mentioned that the CC behavior arises from the intermediate water, which is weakly hydrogen-bonded to the polymer or the surrounding water molecules.^19−21^

Although the CC behavior of protein-stabilizing ILs was speculated to arise from a similar hydrogen-bonding network of water molecules in biocompatible polymers, the structure of the water molecules around the ILs and the nature of their interaction is yet to be explained clearly. The difficulty in elucidating the IL-water interaction could be owed to the complex and competitive hydrogen bonding between cations, anions, and water molecules.^22^ Furthermore, the hydration state of the ions could also differ based on their structure; for instance, the ion’s hydrogen-bonding capacity,^23,24^ hydrophobicity,^25,26^ and charge locality.^27,28^

The IL–water interaction plays a dominant role in stabilizing proteins. Nevertheless, why do only particular Hy ILs show protein-stabilizing capability? How does this IL–water interaction differ from the protein-denaturing Hy ILs? We hypothesize that protein-stabilizing ILs have a unique intermolecular interaction with water molecules. In this study, we attempt to explain the hydration state of protein-stabilizing ILs by drawing connections between IL–water intermolecular interaction and the water structure around diverse Hy ILs at room temperature.

## 2 Materials and Methods

### 2.1 Preparation of Hy ILs

**Figure 1:**
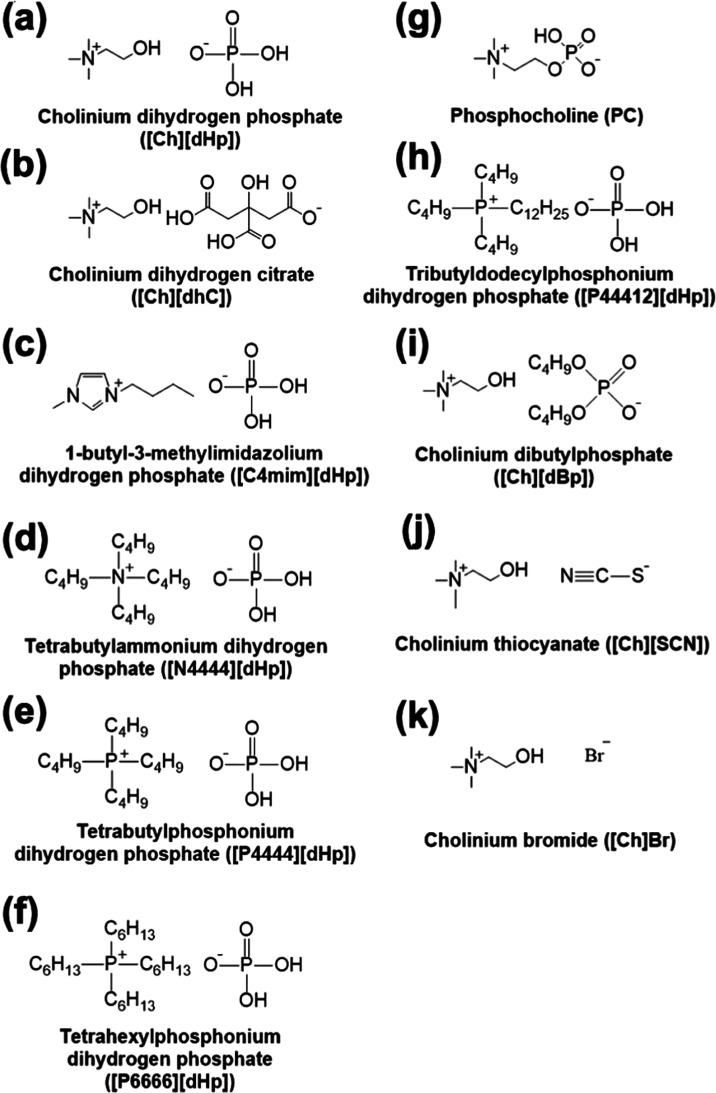
Chemical structures of the ILs investigated in this study (a–k).

In this study, we analyzed the hydration state of choline dihydrogen phosphate ([Ch][dHp]), choline dihydrogen citrate ([Ch][dhC]), 1-butyl-3-methylimidazolium dihydrogen phosphate ([C4mim][dHp]), phosphocholine (PC), tetrabutylphosphonium dihydrogen phosphate ([P4444][dHp]), tetrahexylphosphonium dihydrogen phosphate([P6666][dHp]), tributyldodecylphosphonium dihydrogen phosphate ([P44412][dHp]) and tetrabutylammonium dihydrogen phosphate ([N4444][dHp]), choline dibutylphosphate ([Ch][dBp]), choline bromide ([Ch]Br), and choline thiocyanate ([Ch][SCN]). The chemical structure of the ILs used in this study is depicted in Figure 1; their protein-stabilizing capability and CC behavior are summarized in Table 1. The ILs were synthesized according to the literature.^12,29,30^ ILs were identified by 1H NMR and electrospray ionization mass spectrometry (ESI-MS) or elemental analysis. [P4444][dHp] ESI-MS ESI^+^*m*/*z* 259.25 (P4444^+^), ESI^–^ 78.96 (dHp^–^-H_2_O),^1^H NMR (CDCl_3_, 400 MHz): d 0.80 (s, 12H), d 1.39 (s, 15H), d 2.20 (s, 8H), [N4444][dHp] ESI-MS ESI^+^*m*/*z* 242.28 (N4444^+^), ESI^–^ 253.80 (3 dHp^–^-2H_2_O), ^1^H NMR (CDCl_3_, 400 MHz): d 0.84 (s, 12H), d 1.31 (s, 8H), d 1.50 (s, 8H), d 3.16 (s, 8H), [P44412][dHp] ESI-MS ESI^+^*m*/*z* 371.38 (P44412^+^), ESI^–^ 96.96 (dHp^–^), ^1^H NMR (CDCl_3_, 400 MHz): d 0.89 (m, 12H), d 1.21 (s, 18H), d 1.49 (s, 14H), d 2.27 (s, 8H), [P6666][dHp] ESI-MS ESI^+^*m*/*z* 371.37 (P6666^+^), ESI^–^ 253.80 (3 dHp^–^-2H_2_O) ^1^H NMR (CDCl_3_, 400 MHz): d 0.84 (s, 12H), d 1.28 (m, 32H), d 2.46 (s, 8H). The ILs were mixed with water at 3:1, 7:1, and 15:1 molar ratios of water to IL.

**Table 1:** Protein-Stabilizing Capability and CC Behavior of the Hy ILs Used in This Study

Hy IL	CC (water/IL molar ratio)*	protein stabilization	refs
[Ch][dHp]	Yes (7:1)	Yes	(13)
[Ch][dhC]	Yes (7:1)	Yes	(13)
[C4mim][dHp]	Yes (12:1)	Yes	(13)
[N4444][dHp]	Yes (7:1)	Yes	(27)
[P4444][dHp]	Yes (7:1)	Yes	(27)
[P6666][dHp]	Yes (7:1)	Yes	(27)
PC	Yes (7:1)	Yes	(13)
[P44412][dHp]	Yes (15:1)	Yes	(27)
[Ch][dBp]	No	No	(13)
[Ch][SCN]	No	No	(29)
[Ch]Br	No	No	(29)

* The molar ratio at which the CC was observed is represented in brackets.

### 2.2 ATR-IR Measurement

The IR spectra of all of the Hy ILs were obtained from a single-reflection ATR accessory (“ATR PRO ONE”, JASCO, Japan), assembled in an FT/IR-4600 Fourier transform infrared (FTIR) spectrometer (JASCO, Japan) equipped with a DLATGS detector and a Ge/KBr beam splitter. A tiny droplet of the Hy IL was pipetted on top of the diamond ATR prism and sealed with a lid to mitigate the evaporation of water. All measurements were performed at room temperature with a continuous flow of dry N_2_ gas. The spectra were averaged over 150 scans with a resolution of 4 cm^–1^ (1 cm^–1^ in the data step). The IR absorption spectrum was processed with Spectral Manager (JASCO, Japan) and analyzed with Igor Pro (Wavemetrics, USA). We confirmed that the conditions of the measurements and spectral processing can reveal the detailed structural features (number of peaks and peak positions) of the spectra.

### 2.3 Curve Fitting Analysis

The IR spectra of the OH stretching band is broad, which results from the intermolecular vibrational dynamics of stretching vibrations of water molecules at various hydrogen-bonding state. In the case of Hy ILs, the broadness arises from the mixing of the CH stretching mode and the Fermi resonance in the OH stretching region of the IR spectra. To deconvolute this broad OH stretching band, we performed Gaussian deconvolution. In this section, we will demonstrate the curve fitting analysis using Hy [Ch][dHp] 7:1 (protein-stabilizing Hy IL) and [Ch]Br 7:1 (protein-denaturing Hy IL). The remaining curve fitting results of the OH stretching band of Hy ILs are included in the Supporting Information (Figures S1–S3)

**Figure 2:**
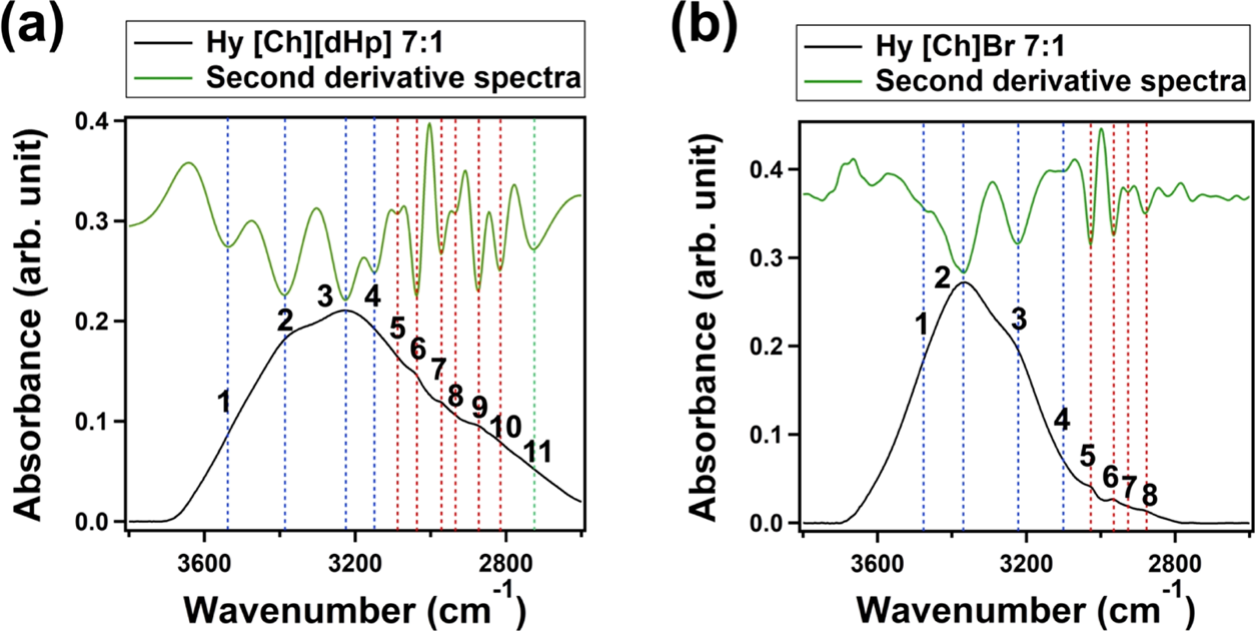
Number of peaks and their positions identified from the second derivative analysis of the OH stretching bands of Hy [Ch][dHp] 7:1 and Hy [Ch]Br 7:1.

Second derivative analysis was performed on the OH stretching bands to identify the number of peaks and their positions (Figure 2). The negative peaks of the second derivative spectra in the 3700–3100 cm^–1^ appear to be broader than the negative peaks in the 3100–2800 cm^–1^ region.

The negative peaks in the 3700–3100 cm^–1^ region could be correlated with the OH stretching peaks, and the negative peaks in the 3100–2800 cm^–1^ region could be correlated with the CH stretching peaks.

**Figure 3:**
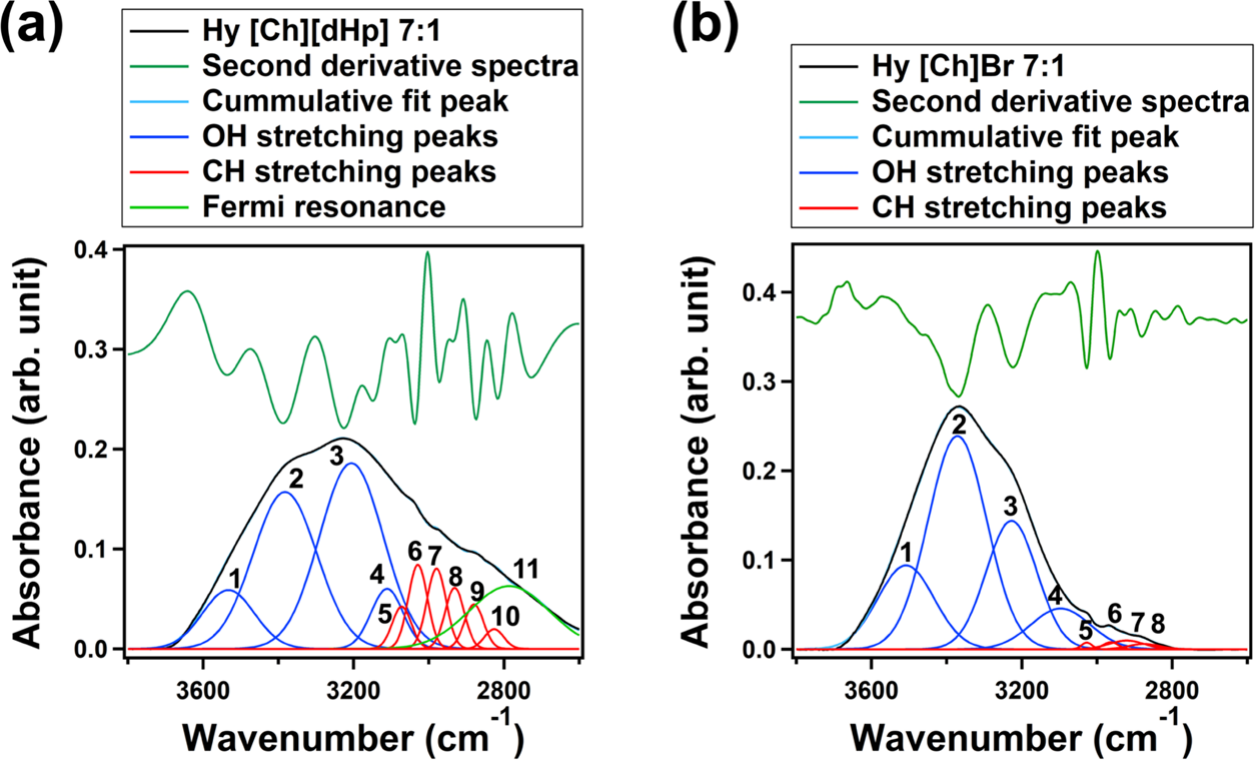
The number of peaks and their positions identified from the second derivative analysis of the OH stretching bands of Hy [Ch][dHp] 7:1 and Hy [Ch]Br 7:1.

Curve fitting of the OH bands of the Hy ILs was performed with a Gaussian function using the peak positions identified from the second derivative spectra (Figure 3). However, the peaks in the CH stretching region were fixed as per the second derivative spectra. Fixing the CH stretching peaks mitigated the fluctuation of fitting results. Together with the error during fitting, the peak areas fluctuated within 4%.

The protein-stabilizing Hy ILs show a relatively broader OH stretching band compared to the protein-denaturing Hy ILs. This broadness of the OH band arises from the Fermi resonance caused by the mixing of the overtone of the out-of-plane bending vibration mode of the P–OH group of the [dHp]^−^ anion with the CH stretching and OH stretching bands of the cation.^31^ The assignments of the peaks are represented in Table 2.

**Table 2:** Assignment of the Gaussian Peaks of Hy ILs in the 3700–2700 cm^–1^ Region of IR Spectra

IR peak frequencies (cm^–1^)	assignments
3700–3000	OH stretching band
3090–3070	CH asymmetric stretching mode of (N)CH_3_ group
3040–3020	CH symmetric stretching mode of (N)CH_3_ group
2980–2960	CH asymmetric stretching mode of CH_3_ group
2940–2920	CH symmetric stretching mode of CH_3_ group
2880–2870	CH asymmetric stretching mode of CH_2_ group
2850–2820	CH symmetric stretching mode of CH_2_ group
2800–2700	Fermi resonance from the overtone of P–OH out-of-plane bending peak of the [dHp] anion mixing with the CH and OH stretching bands

## 3 Results and Discussion

Although several Hy ILs have been reported to show protein-stabilizing capability, the intermolecular interaction between water and IL has yet to be elucidated clearly. This study aims to probe into the intermolecular interaction between several Hy ILs ionic liquids and analyze its effect on the hydrogen-bonding network of the water molecules. By investigating the intermolecular interaction between IL and water, we try to suggest an explanation for the protein-stabilizing capability of specific Hy ILs.

### 3.1 OH Stretching Band

#### 3.1.1 Hydrogen-Bonding Network of Water Molecules Is Relatively Less Perturbed in Protein-Stabilizing Hy ILs Than in Protein-Denaturing Hy ILs

**Figure 4:**
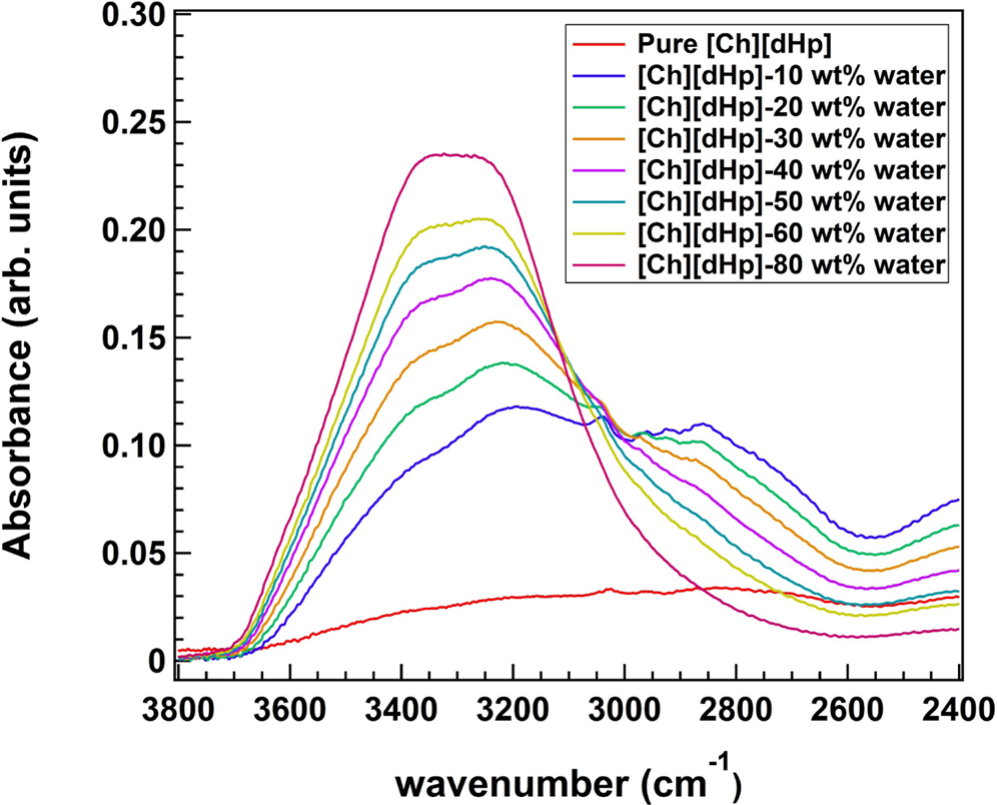
IR spectra of the stretching band of [Ch][dHp] with various water contents.

The OH stretching bands of the [Ch] cation and [dHp] anion are mixed with the OH stretching band of water. However, the OH stretching band of the pure ionic liquid (IL) (red plot) was weak in absorbance and broad, spanning from ∼3700 to ∼2000 cm^–1^ (Figure 4). Hence, the dominant contribution to the OH stretching band in hydrated (Hy) [Ch][dHp] comes from water. Therefore, we concluded that the OH stretching band dominantly arises from the stretching vibration of the water molecules.

The OH stretching band is considered sensitive to the variation of the hydrogen-bonding network of water molecules.^32−34^ The OH stretching band of the Hy ILs gives us an idea about the structure of water molecules in the hydration shell of ions.^35−37^ We investigated the OH stretching band of water structure in the most biocompatible IL–water composition, 7:1 molar ratio, as seven water molecules per ion pair was reported to be the threshold hydration state to ensure biological activity.^38^

**Figure 5:**
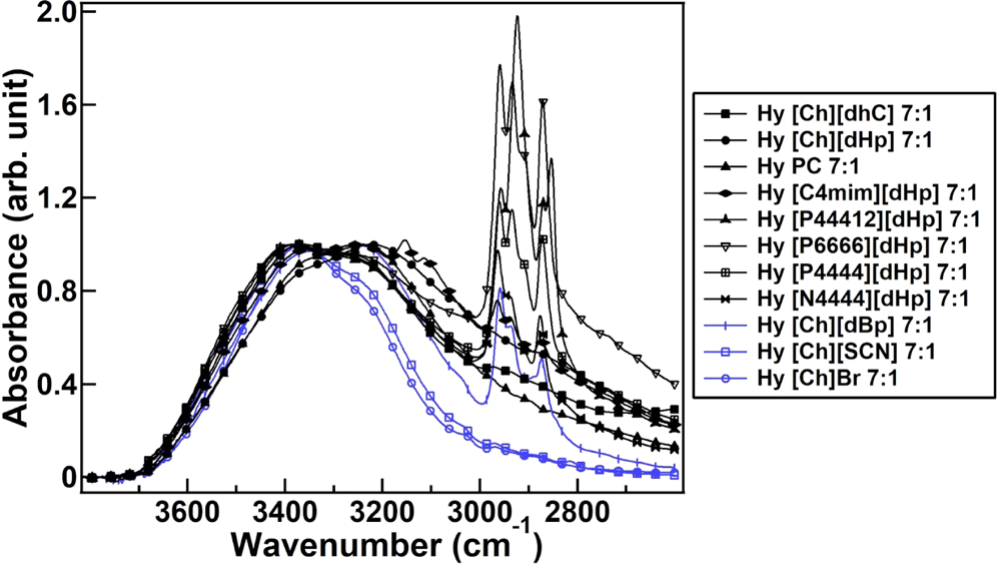
ATR-IR spectra of the OH stretching band of protein-stabilizing Hy ILs (black line) and protein-denaturing Hy ILs (blue line) at a 7:1 molar ratio. The OH stretching bands of the spectra were height-normalized at the OH stretching region for comparison.

**Figure 6:**
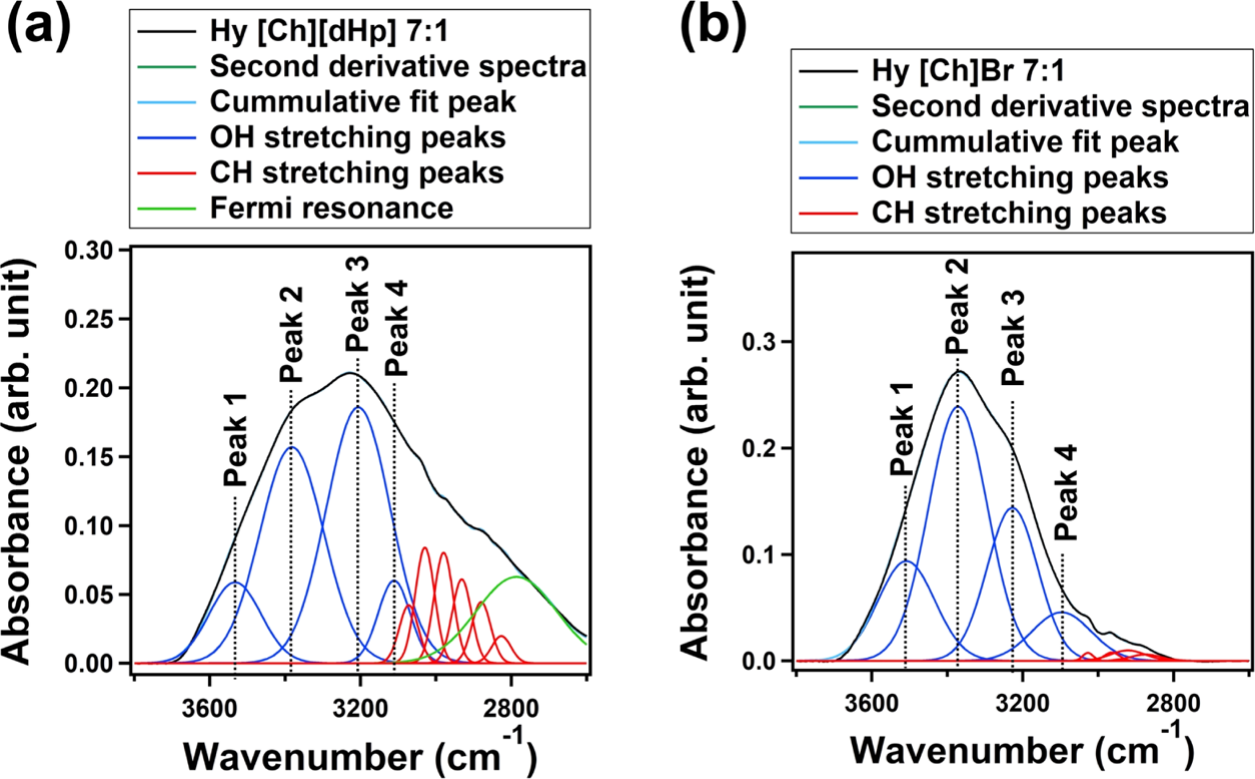
OH stretching band of (a) Hy [Ch][dHp] 7:1 (protein-stabilizing IL) and (b) Hy [Ch]Br 7:1 (protein-denaturing IL) decomposed into four Gaussian peaks. The number of peaks and their positions were identified from the second derivative analysis.

The ATR-IR spectra of the OH stretching band of Hy ILs at a 7:1 molar ratio are illustrated in Figure 5. To analyze the difference in the hydration structure of protein-stabilizing and protein-denaturing Hy ILs, the OH stretching band was deconvoluted into four Gaussian peaks. The number of peaks and their peak positions were evaluated from the second derivative analysis. Figure 6 demonstrates the Gaussian peak deconvolution of the OH band of Hy [Ch][dHp] 7:1 (a protein-stabilizing Hy IL) and Hy [Ch]Br 7:1 (a protein-denaturing Hy IL). The remaining deconvolution results of the Hy ILs and pure water (neat water) are included in the Supporting Information (Figures S1–S3).

**Figure 7:**
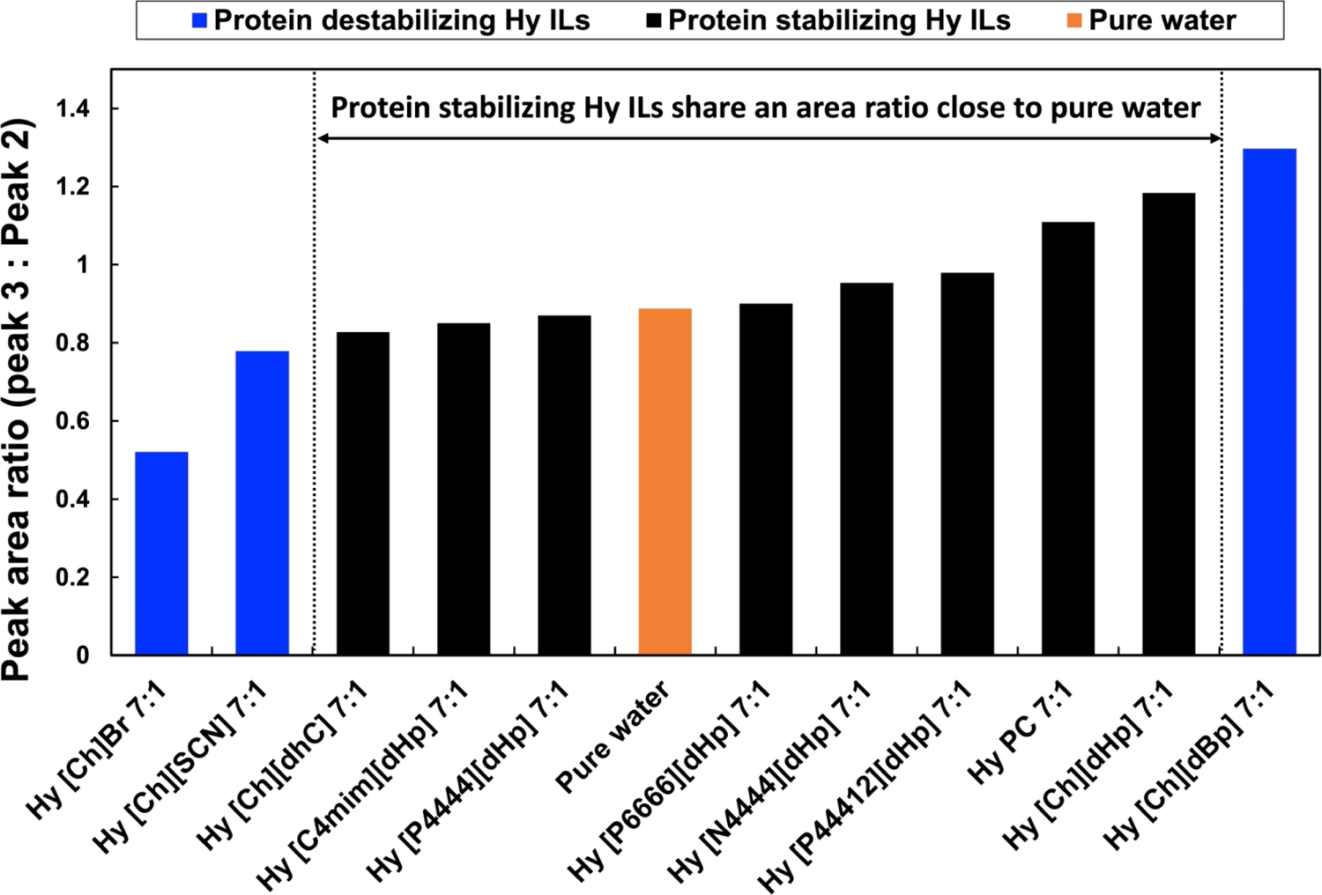
Summarized peak area ratio of the prominent Gaussian peaks of the OH stretching band of pure water and Hy ILs at a 7:1 molar ratio.

The OH band has two prominent peaks at ∼3200 cm^–1^ (peak 3) and ∼3400 cm^–1^ (peak 2). These two peaks represent the two most abundant local hydrogen-bonding networks present within the water clusters. The peak at around ∼3200 cm^–1^ was previously associated with strongly hydrogen-bonded water molecules, and the peak at around ∼3400 cm^–1^ was associated with weakly hydrogen-bonded water molecules.^32,39^ We used these two prominent peaks to analyze the difference in the trend of the OH stretching band among the Hy ILs. We calculated the area of the Gaussian peaks 2 and 3 of the OH stretching band of Hy IL 7:1 and pure water. The area ratios (peak 3: peak 2) of the Gaussian components are summarized in Figure 7. The peak area ratio of pure water was 0.89, and the peak area ratio of protein-stabilizing Hy ILs ranged from 0.83 to 1.18.

Moreover, in protein-denaturing Hy ILs, Hy [Ch]Br 7:1 and Hy [Ch][SCN] 7:1 had area ratios of 0.52 and 0.78, while Hy [Ch][dBp] 7:1 showed an area ratio of 1.30. From Figure 7, it is evident that there is a window within which the protein-stabilizing ILs share an area ratio close to that of pure water (0.89). Beyond this window, the area ratios of the protein-denaturing ILs are distant from that of pure water.

Suppose the protein-stabilizing Hy ILs showed an area ratio close to that of pure water. In that case, this means that the proportion of strongly hydrogen-bonded water molecules to weakly hydrogen-bonded water molecules within the Hy IL is relatively similar to that of pure water. In other words, in the protein-stabilizing Hy ILs, to a certain extent, the hydrogen-bonding network of the water molecules was unperturbed.^40^ In the case of protein-denaturing Hy ILs, the area ratio is further away from that of pure water. This finding may indicate that the local hydrogen-bonding network of water molecules was severely disrupted in the protein-denaturing IL. For example, Hy [Ch]Br 7:1 and Hy [Ch][SCN] 7:1 had an area ratio of 0.52 and 0.78, respectively, much lower than that of the pure waters, 0.89. A lower area ratio means that the number of water molecules engaged in weak hydrogen bonding with anions is greater, which reduces the number of strongly hydrogen-bonded water molecules.^35,36,41^ We also investigated the correlation between the area ratios of other peaks and the stabilization of proteins. However, we did not find a clear correlation; the summary of the peak areas is included in the Supporting Information (Figure S4).

The Br^–^ and [SCN]^−^ are large and aprotic anions; the increased surface area and the lack of a hydrogen-bonding site increase the surface interaction with many water molecules.^42^ This surface interaction of water molecules with anions causes the hydrogenic part of the water molecule to face toward the anion, which prevents hydrogen-bonding interaction with neighboring water molecules.^43^ On the other hand, Hy [Ch][dBp] 7:1, also a protein-denaturing IL with an area ratio (1.30), is much larger than that of the protein-stabilizing IL. The component associated with the strongly hydrogen-bonded water molecules was more significant than the weakly hydrogen-bonded water molecules. This interaction is unlike the native hydrogen-bonding interaction of pure water, and hence the area ratio of Hy [Ch][dBp] 7:1 falls outside of the window. One possible explanation for this behavior could be that the weak interaction of water molecules with the [dBp]^−^ anion and its long alkyl chain facilitates a favorable configuration of the water cluster.^25,37^ The pocket created by the anion’s hydrophobic domains may increase the water–water aggregation. Hence, the component of strongly hydrogen-bonded water is the dominant local hydrogen-bonding state of the Hy [Ch][dBp] system. The area ratios of the two components indicate that in protein-stabilizing ILs, the native hydrogen-bonding state of water molecules is relatively less perturbed than in protein-denaturing ILs.

Similar phenomena were observed in biocompatible zwitterionic polymers such as poly(2-methacryloyloxyethyl phosphorylcholine) (PMPC),^44^ poly[*N*,*N*-dimethyl-*N*-(3-sulfopropyl)-3′-methacrylamidopropanaminium inner salt] (poly(SPB)),^45^ and poly(2-methoxyethyl acrylate) (PMEA).^46^ The vibrational spectra of the water molecules in the vicinity of the polymers mentioned above were reported to be similar to the spectrum of pure water.^47^ It was also mentioned that the high blood compatibility of the zwitterionic copolymer of MPC and butyl methacrylate (BMA) [poly(MPC-*r*-BMA)], and PMEA may have arisen from the intact hydrogen-bonding network of water in its vicinity.

This unperturbed hydration shell may mediate the electrostatic interaction between the ions of the ILs and the protein molecules, preserving the structure and function of protein molecules. The absence of the pure water-like hydrogen-bonding network in Hy ILs may result in an imbalanced interaction between the ions, water molecules, and protein molecules. That is, the ions may directly interact with the charged moiety of the protein and denature it.

### 3.2 HOH Bending Mode

#### 3.2.1 Protein-Stabilizing ILs Have a Stronger Electric Field Interaction with Water Molecules Than Protein-Denaturing ILs

Just like the IR OH stretching band, the HOH bending peak is also equally indicative of the hydrogen-bonding interaction of the water molecules. Furthermore, the line shape of the bending mode is also subjected to less vibrational coupling effects from the water molecules’ intra- and intermolecular bending vibrations compared to the OH stretching band.^48^ The vibrational frequency and the peak shape of the HOH bending mode spectrum could be used to probe into the electrostatic interaction of the water molecules and ions.^49^

**Figure 8:**
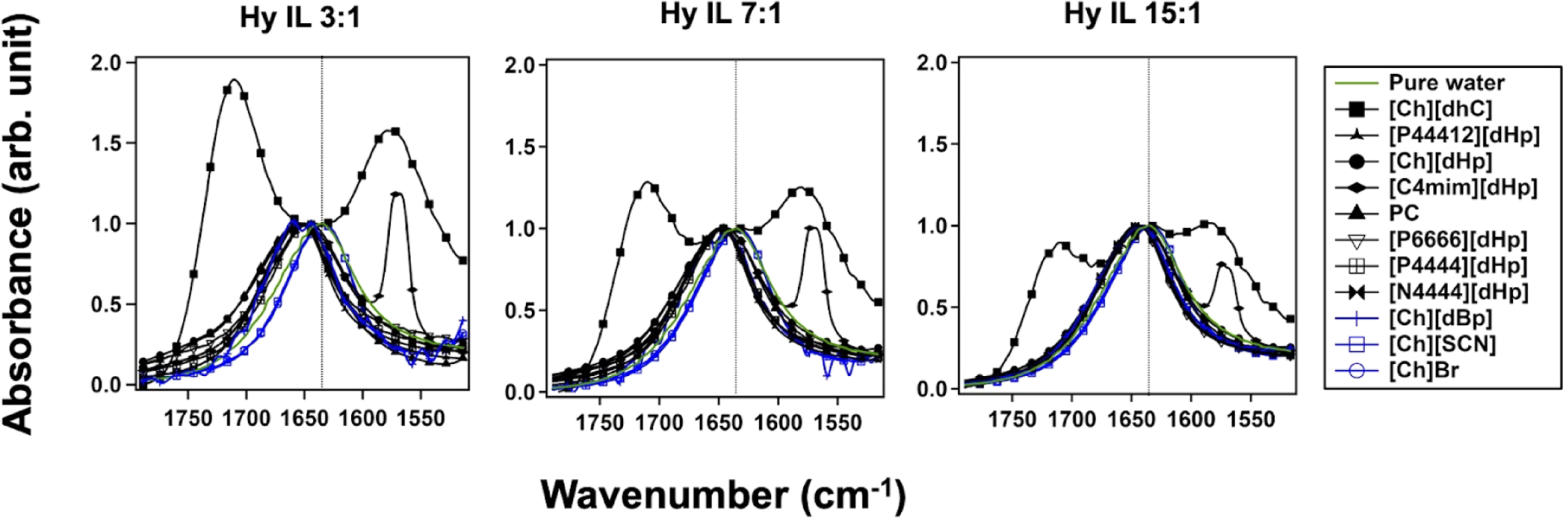
Spectra of HOH bending mode of Hy ILs at (a) 3:1, (b) 7:1, and (c)15:1 molar ratios. The spectra were height-normalized for comparison. The dashed line indicates the peak maxima position of pure water’s HOH bending spectrum.

**Figure 9:**
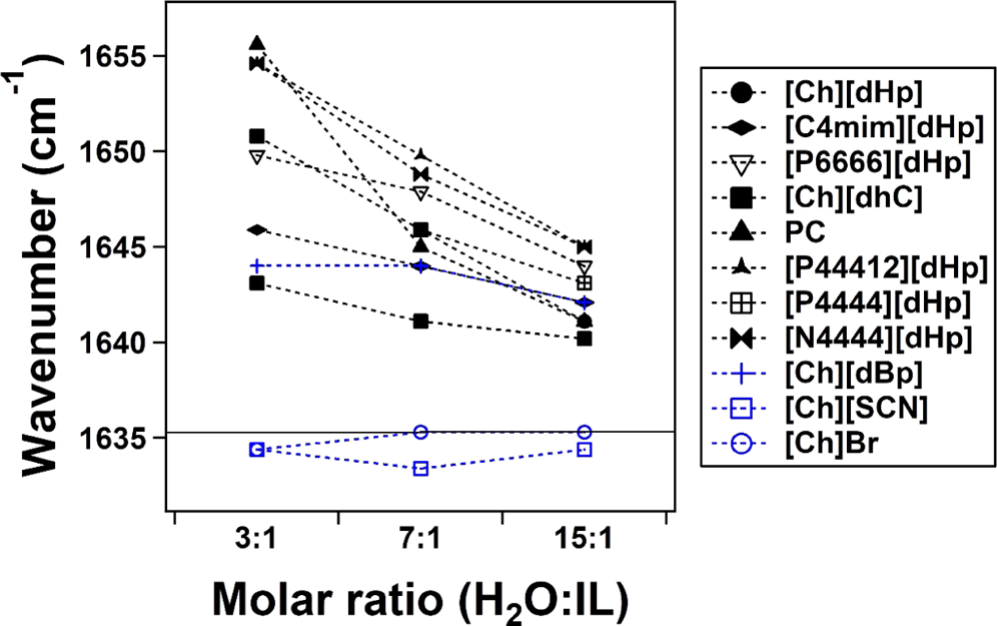
Change in the peak top position of HOH bending spectra of Hy ILs with respect to increased water content. Black color indicates protein-stabilizing Hy ILs, and blue color represents protein-denaturing Hy ILs. The horizontal solid line indicates the peak top position of pure water’s HOH bending spectrum.

Figure 8 illustrates the HOH bending spectra of Hy IL at 3:1, 7:1, and 15:1 molar ratios. The HOH bending mode frequency of pure water was 1635.3 cm^–1^. To analyze the effect of IL on the water molecules, the maxima of the HOH bending peak were plotted as a function of increasing water content (Figure 9).

At a 3:1 molar ratio, the electric field effect of the ions on the water molecules is the strongest due to the relatively low water–water interaction. The HOH bending frequency of all protein-stabilizing Hy ILs at a 3:1 molar ratio is higher than that of pure water (1635.3 cm^–1^), and with further addition of water content, the HOH bending vibration gradually reduced. In the case of protein-denaturing Ils, except for Hy [Ch]dBp (1644 cm^–1^), the HOH bending peak at the 3:1 molar ratio of Hy [Ch]Br and Hy [Ch][SCN] has the same bending vibrational frequency of 1634.4 cm^–1^, lower than that of pure water. But with increasing water content, the frequency shift of the maxima is unlike the protein-stabilizing Ils. For example, the HOH bending peak of Hy [Ch][dBp] 3:1 remains unchanged until the 7:1 molar ratio and reduces to a lower frequency only at the 15:1 molar ratio. An increase in water concentration showed no effect on the vibrational frequency until the 15:1 molar ratio. In Hy Ch Br 3:1, the peak maximum was 1634.4 cm^–1^, and at a 7:1 molar ratio, the maximum shifts to reach the frequency of pure water (1635.3 cm^–1^), and this maximum position remains consistent up to 15:1 molar ratio. In the case of the Ch SCN, the maximum position at 3:1 molar ratio was also 1634.4 cm^–1^, but with increasing water content it reduced to 1633 cm^–1^ at 7:1 molar ratio, and at 15:1, it returned to its initial position at 1634.4 cm^–1^. To summarize, the protein-denaturing Hy Ils showed an HOH bending frequency higher than pure water, while the protein-denaturing Ils showed lower bending vibrational frequency than pure water except [Ch][dBp], and their peak shifts are inconsistent with increasing water concentration. In protein-stabilizing Hy Ils, the HOH bending frequency reduces consistently with increasing water concentration.

The Increase in the HOH bending frequency of the water indicates that the water molecules are subjected to the electric field effect of the protein-stabilizing ILs. The multiple proton donor and acceptor sites in [dHp]^−^ and [dhC]^−^ anions of the protein-stabilizing ILs may enable directional hydrogen bonding with the surrounding water molecules. The directional hydrogen bonding results in a strong electrostatic pull of water molecules’ hydrogen end toward the oxygen of the anion hydrogen-bonding acceptor. This attraction may affect the bending vibrational frequency of the water molecule. As the vibrational bending mode of the water molecule involves the change in the O–H bond angle, during the vibration, the O–H bond pulls away from the anion. However, due to the energy penalty of pulling away from a directional hydrogen bond, the O–H bond vibrates with a slight change in bond angle. Hence, the vibrational frequency is much higher than in pure water. A lower bending vibrational frequency indicates that the electrostatic interaction of water molecules is weaker than that of pure water. [Ch]Br and [Ch][SCN] may have had a structure-breaking effect on the water molecules. That is, the O–H bond of the water molecules is not strongly pulled by neighboring water molecules or the ions. Hence, the bond angle change during bending vibration is significant, resulting in a lower vibrational frequency.^49^

With the further addition of water molecules, the water–water hydrogen-bonding interaction dominates the electric field effect of the ions, and the bending frequency approaches that of pure water. Unlike the other protein-denaturing ILs, Hy [Ch][dBp] 3:1 has a higher frequency of HOH bending vibration, 1644 cm^–1^. With the further addition of water content, the peak maximum does not change until the 7:1 molar ratio. This result could be interpreted such that the water molecules at a 3:1 molar ratio, when interacting with ions, achieve a favorable cluster, and this hydrogen-bonding network remains stable up to a 7:1 molar ratio, after which the water–water hydrogen-bonding interaction dominates the mixtures. As mentioned in the previous discussion for the area ratio of the OH stretching band, this stable water cluster may be enabled by the weak interaction with the [dBp]^−^ anion. The anion weakly attracts the water molecules, and the long alkyl chain may create nonpolar domains with pockets of water clusters, which prevent interaction with other water molecules until sufficient water concentration is reached.

These results also agree with ^1^H NMR chemical shifts of water proton in IL 3:1 reported previously by Nikawa et al.^24^ The ^1^H NMR chemical shift of pure water was 4.8 ppm. A change in the chemical shift of the water proton indicates a change in its electron density. To illustrate, a gain in electron density of water proton results in shielding of the hydrogen atom, which leads to an upfield shift, and a decrease in the chemical shift (<4.8 ppm). On the other hand, the loss of electron density of the water proton results in the deshielding of the hydrogen atom, which leads to a downfield shift, that is, the chemical shift value increase (>4.8 ppm). The protein-stabilizing ILs such as Hy [Ch][dHp] (6.3 ppm), Hy [Ch][dhC] (6.8 ppm), Hy PC (5.8 ppm), Hy [C4mim][dHp] (6.1 ppm) showed a downfield shift. Moreover, the protein-denaturing ILs, [Ch][SCN] (4.2 ppm), showed a corresponding upfield chemical shift, and [Ch][dBp] (4.85 ppm) showed a negligible chemical shift from that of pure water. The downfield shift of the protein-stabilizing ILs ranging from 1 to 2.8 ppm from that of the pure water indicates that the ILs have a strong electric field effect on the water molecules. The formation of directional hydrogen bonds with the anions electrostatically screens the water molecules.^50^ The significant change in the chemical shift may also indicate that the electric field effect of the protein-stabilizing ILs may span over several hydration shells. An upfield chemical shift of the water proton indicates the structure-breaking effect, disrupting the water’s hydrogen-bonding network due to weak interaction with anions.^43^ Our HOH bending results agree with these NMR chemical shift results. We hypothesize that the IR HOH bending peak shifts also represent the ion-dipole and hydrogen-bonding interaction between the IL and water molecules.

#### 3.3.2 Electric Field Effect of Protein-Stabilizing ILs Does Not Disrupt Water’s Hydrogen-Bonding Network As Much As Protein-Denaturing ILs

We consider that there are two reasons for the change in the energy of the HOH bending mode: water-ion and water–water interactions.^50^ The HOH bending peak of pure water could be assumed as consisting of only water–water interaction. Hence, any change in the broadness of the HOH bending peak of Hy ILs could indicate a change in the proportions of the two aforementioned water components.

**Figure 10:**
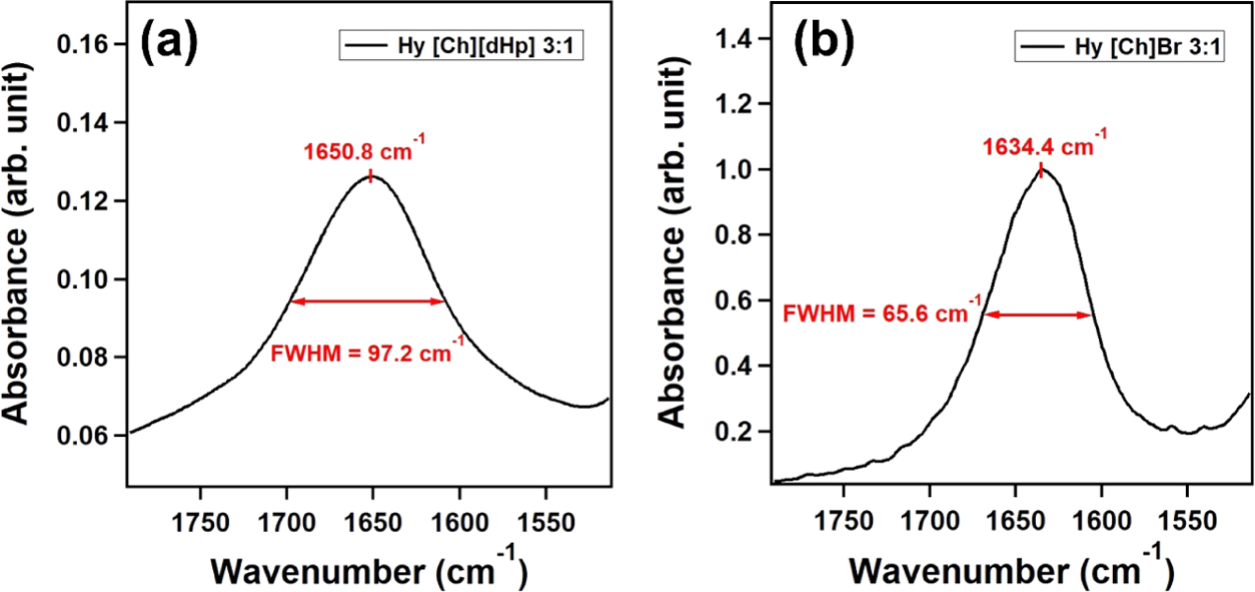
FWHM of HOH bending peaks of (a) Hy [Ch][dHp] 3:1 and (b) Hy [Ch]Br 3:1.

To quantify the broadness, we calculated the full width at half-maximum (FWHM) of the HOH bending mode’s peak of Hy ILs 3:1, where the electric field effect of the IL on the water is dominant. Figure 10 demonstrates the calculated FWHM of [Ch][dHp], protein-stabilizing IL, and [Ch]Br, a protein-denaturing IL. The remaining FWHM results of the Hy ILs are included in the Supporting Information (Figures S5 and S6).

**Figure 11:**
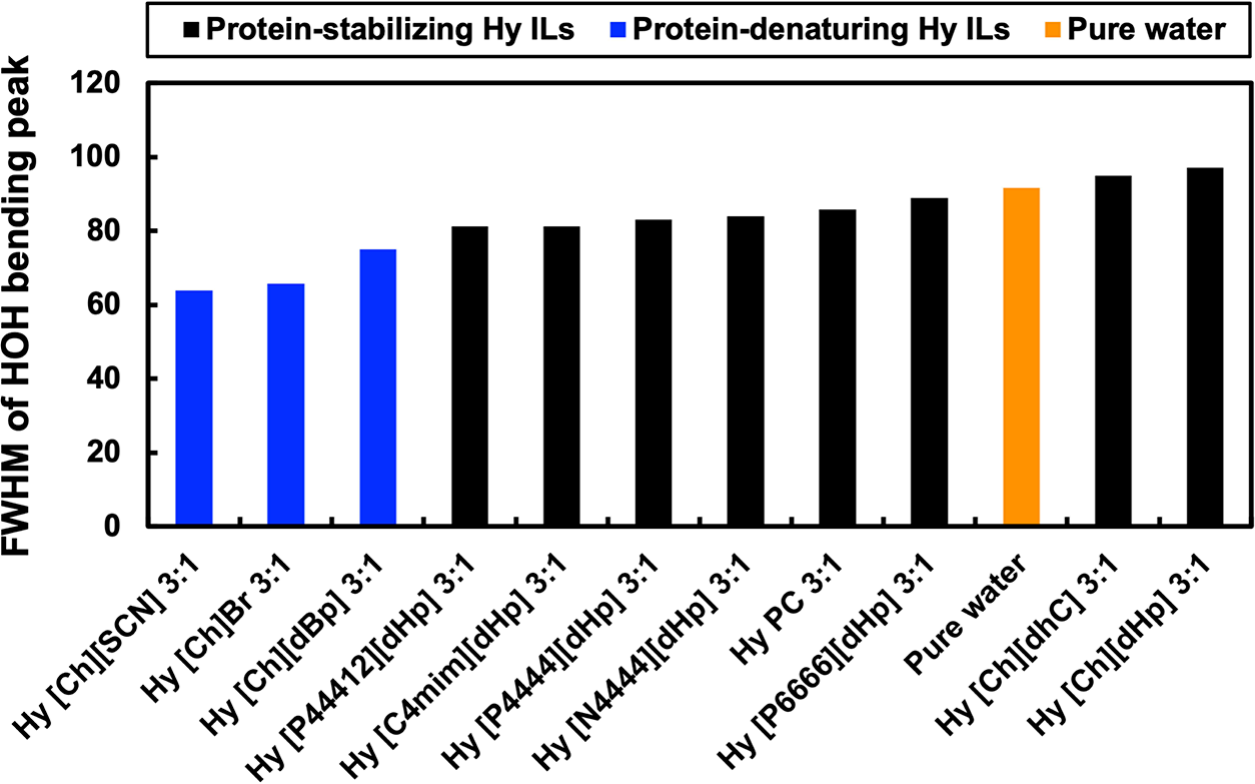
FWHM of the HOH bending peaks of Hy ILs at a 3:1 molar ratio and pure water.

From the Gaussian peak fitting, the FWHM values of all of the Hy IL 3:1 and pure water were calculated and are summarized in Figure 11. The FWHM of the HOH bending mode’s peak of protein-stabilizing Hy ILs is less than that of protein-denaturing ILs. Pure water has an FWHM of 85 cm^–1^, the protein-stabilizing ILs showed an FWHM ranging between 80 and 98 cm^–1^, and the protein-denaturing ILs showed an FWHM ranging from 60 to 78 cm^–1^. In protein-stabilizing Hy ILs, the FWHM values of the HOH bending peaks were in close range to that of pure water. In protein-denaturing Hy ILs, the FWHM values of HOH bending peaks were smaller than those of protein-stabilizing Hy IL and pure water.

In pure water, the broadness of the HOH bending peak arises from the continuous distribution of hydrogen-bonding strength and vibrational coupling of the water molecules.^51^ Hence, a reduction in the FWHM of HOH bending peak in protein-denaturing Hy ILs indicates that the number of water molecules engaging in pure water-like water–water hydrogen bonding within the hydration shell may have been reduced.^52^ On the other hand, the protein-stabilizing ILs show a relatively broader FWHM. This broadness could mean that despite the strong electric field effect of ions, the distribution of hydrogen-bonding strength of the surrounding water molecules is still comparable to that of pure water. Even though the maxima of the HOH bending peaks were blue-shifted due to the electric field effect of ions, the peaks still retained components from the water–water hydrogen-bonding interaction. From these results, we can speculate that even though the protein-stabilizing ILs electrostatically screen the water molecules, this electric field effect does not strip the water molecules from its water–water hydrogen-bonding interaction.

Ahmed et al. reported that the FWHM of the HOH bending mode’s peak of the Raman spectra of aqueous NaCO_3_ was also as large as pure water’s.^52^ They performed Raman spectroscopy and multivariate curve resolution (MCR) analysis to deconvolute the HOH bending spectra into two components. One component corresponds to the vibrational response of the water molecules perturbed by the ions, and the other component corresponds to water in a pure water-like state. The FWHM of the ion-perturbed water spectra in aqueous NaCO_3_ was almost the same as that of the pure water spectra. This broadness of the HOH band was associated with a greater distribution of the hydrogen-bonding strength of the water molecules in the hydration layer. The authors also suggested that in aqueous NaCO_3_, the water molecules exist in two types of hydrogen bonding. In the first hydration shell, water molecules are strongly hydrogen-bonded to the oxygen atom of the CO_3_^2–^ anion. In the second hydration shell, the water molecules are hydrogen-bonded to other water molecules by slightly weaker hydrogen bonds than in the first hydration shell. But still stronger than pure water’s hydrogen-bonding strength. We speculate that the water molecules in the protein-stabilizing ILs may also exist in such hydrogen-bonding states. The strong electric field effect of the protein-stabilizing ILs on water molecules and the retainment of pure water-like hydrogen-bonding interaction in the hydration shells may play an essential role in stabilizing protein molecules.

## 4 Conclusions

This study used ATR-IR spectroscopy to demonstrate the complex intermolecular interaction between protein-stabilizing ionic liquid and water molecules. By investigating the components of the IR OH stretching band of the Hy ILs, we observed that the native hydrogen-bonding network of the water molecules is less perturbed in the hydration shells around the protein-stabilizing ILs compared to protein-denaturing ILs. From the peak maximum and the FWHM of HOH bending pending peak of the Hy ILs, we found that the water molecules are subjected to a strong electric field by the protein-stabilizing ILs. Furthermore, this electric field effect of the ionic liquids does not disrupt the water–water hydrogen-bonding interaction. On the other hand, protein-denaturing ILs have a weak electric field, and this weak interaction destabilizes the water’s hydrogen-bonding network. We believe that the direct hydrogen bonding of the ILs with water molecules and the strong electric field of the ions lasting several hydration shells while maintaining the relatively unperturbed hydrogen-bonding network of the water molecules play an essential role in protein stabilization.

Although the protein stabilization capability of aqueous salt solutions has been discussed in terms of the Hofmeister series of ions, the protein stabilization mechanisms discussed in this work for Hy ILs may not correlate with aqueous salt solutions. The protein stabilization or denaturation experiments by aqueous salt solutions were carried out at low salt concentrations (e.g., 1–30 wt % of NaCl in water). On the other hand, the concentration of the ILs used in this work is very high (e.g., 50–80wt % of [Ch][dHp] in water). Therefore, protein stabilization and denaturation mechanisms can differ between Hy ILs and the Hofmeister series of ions.

Our findings here will contribute to the design of protein-stabilizing ILs in the future. To quantify the electrostatic screening effect and the strength of hydrogen bonds between ions and water molecules, we will combine the NMR chemical shifts and the IR HOH bending peak shifts of Hy ILs. This work will be published elsewhere.

## Supporting Information Available

Gaussian deconvolution of the OH stretching band of pure water and Hy ILs (Hy [Ch][dhC] 7:1, Hy [C4mim][dHp] 7:1, Hy PC 7:1, Hy [P4444][dHp] 7:1, Hy [P6666][dHp] 7:1, Hy [P44412][dHp] 7:1, Hy [N4444][dHp] 7:1, Hy [Ch][dBp] 7:1, Hy [Ch][SCN] 7:1) (Figures S1–S3); area ratios of the four Gaussian peaks of the OH stretching band of pure water and the aforementioned Hy ILs at a 7:1 molar ratio (Figure S4); and full width at half-maxima of HOH bending peaks of pure water and the Hy ILs at a 3:1 molar ratio (Figures S5 and S6) (PDF)
