# Supplementary material for: Investigation of Hydration States of Ionic Liquids by Fourier Transform Infrared Absorption Spectroscopy: Relevance to Stabilization of Protein Molecules
Source: Langmuir. 2023 Feb 8;39(7):2558–68. doi: 10.1021/acs.langmuir.2c02851 (PMC9948542; doi:10.1021/acs.langmuir.2c02851)
Supplement: Supplementary file 1 — la2c02851_si_001.pdf [file la2c02851_si_001.pdf]

Supporting information

Investigation of hydration states of ionic liquids by Fourier transform infrared absorption spectroscopy: relevance to stabilization of protein molecules

Navin Rajapriya Inbaraj[1], Subin Song[1], Ryongsok Chang[1], Kyoko Fujita[2,*], Tomohiro Hayashi[1,*]

[1] Department of Materials Science and Engineering, School of Materials Science and Chemical Technology, Tokyo Institute of Technology, 4259 Nagatsuta-cho, Midori-ku, Yokohama-shi, Kanagawa-ken 226-8502, Japan.

[2] Department of Pathophysiology, Tokyo University of Pharmacy and Life Sciences, 1432-1 Horinouchi, Hachioji, Tokyo 192-0392, Japan.

[*]kyokof@toyaku.ac.jp, tomo@mac.titech.ac.jp

Table of contents:

Content	Page No.
Gaussian deconvolution of OH stretching band of Hy IL at 7:1 molar ratio and pure water (Figure S1-S3)	S2-S4
Peak area ratios of the Gaussian peaks of the OH stretching band of pure water and Hy ILs at a 7:1 molar ratio (Figure S4)	S5
Full width half maxima (FWHM) of HOH bending peak of Hy IL at 3:1 molar ratio and pure water (Figure S5-S6)	S6-S7

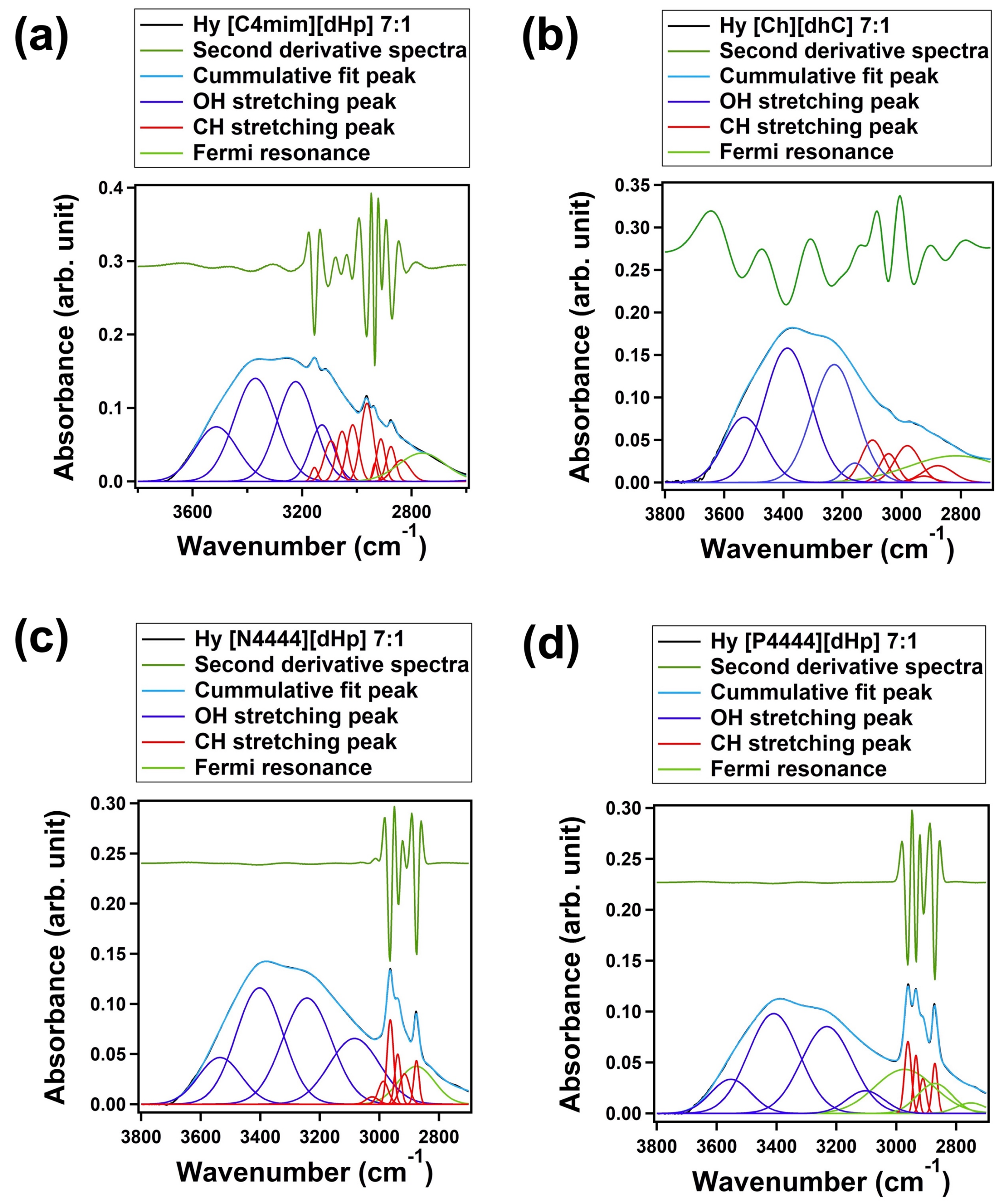

Figure S1. The Gaussian deconvolution of the IR OH stretching band of (a) Hy [C4mim][dHp], (b) Hy [Ch][dhC], (c) Hy [N4444][dHp], and (d) Hy [P4444][dHp] at 7:1 molar ratio. The number of peaks and their positions were identified from the second derivative analysis.

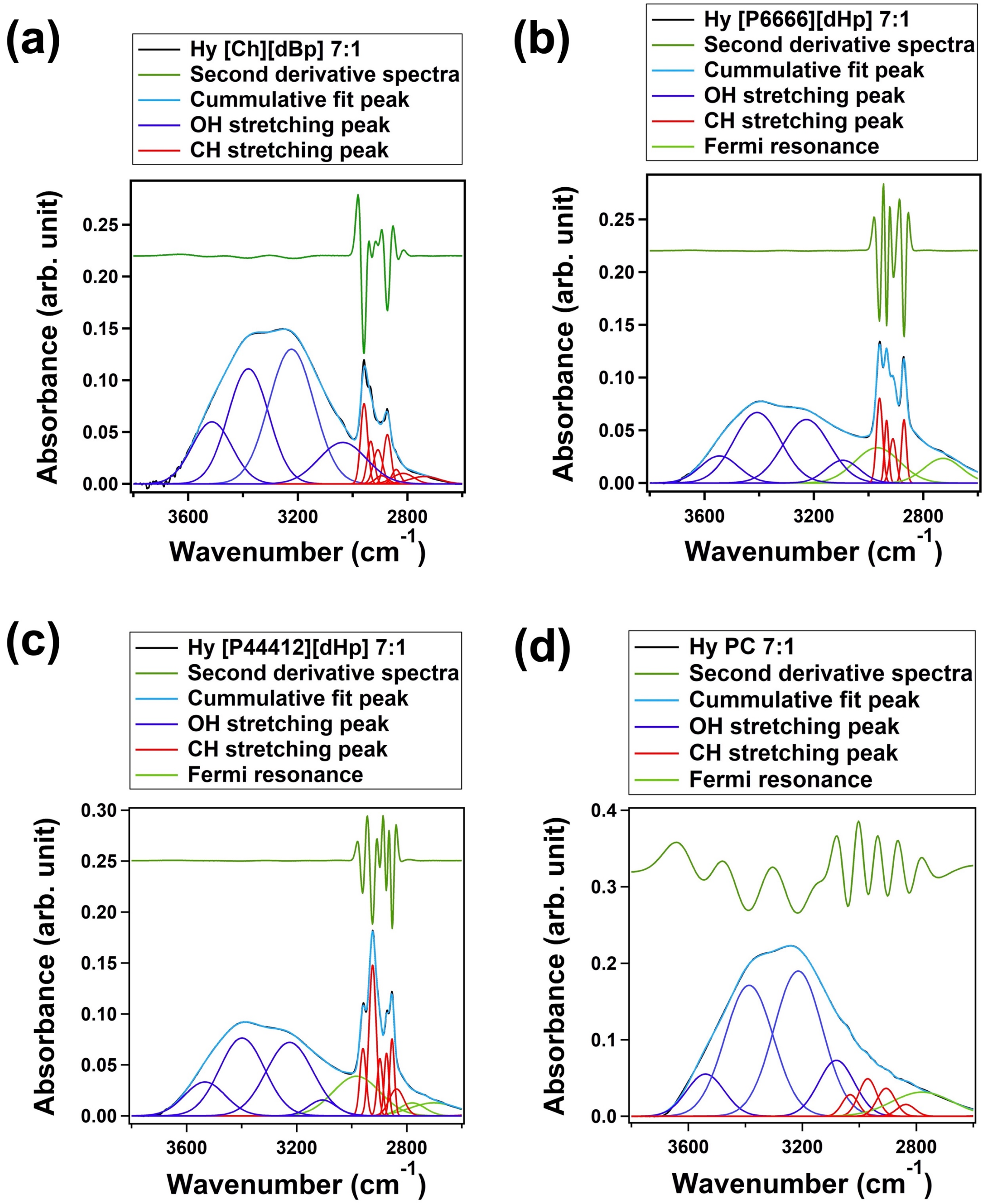

Figure S2. The Gaussian deconvolution of the IR OH stretching band of (a) Hy [Ch][dBp], (b) Hy [P6666][dHp], (c) Hy [P44412][dHp], and (d) Hy PC at 7:1 molar ratio. The number of peaks and their positions were identified from the second derivative analysis.

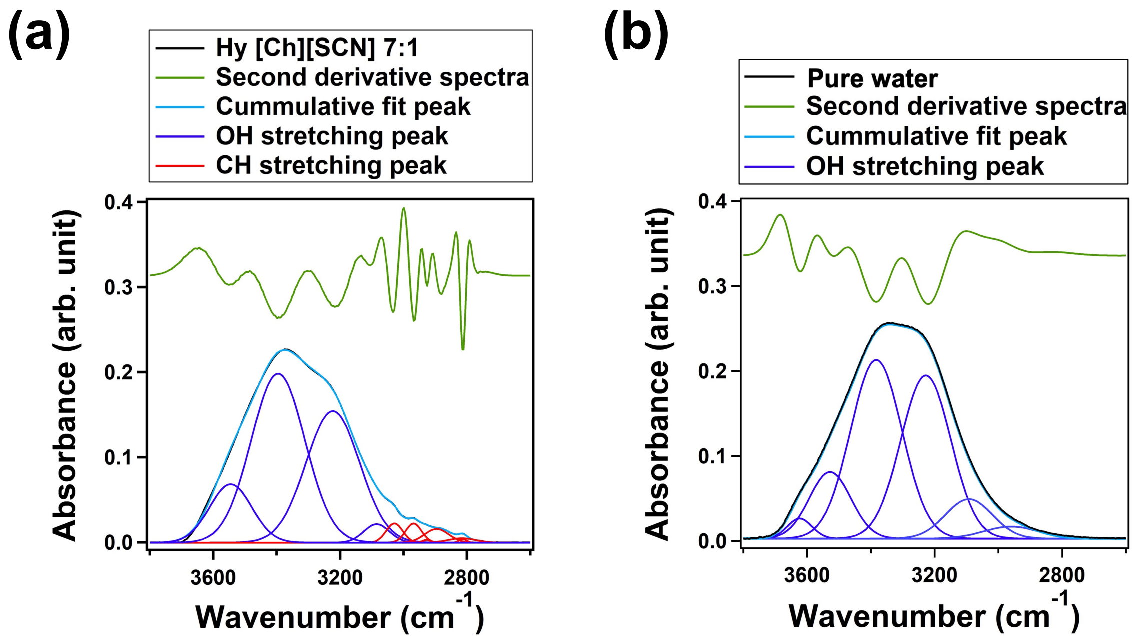

Figure S3. The Gaussian deconvolution of the IR OH stretching band of (a) Hy [Ch][SCN] at 7:1 molar ratio and (b) pure water. The number of peaks and their positions were identified from the second derivative analysis.

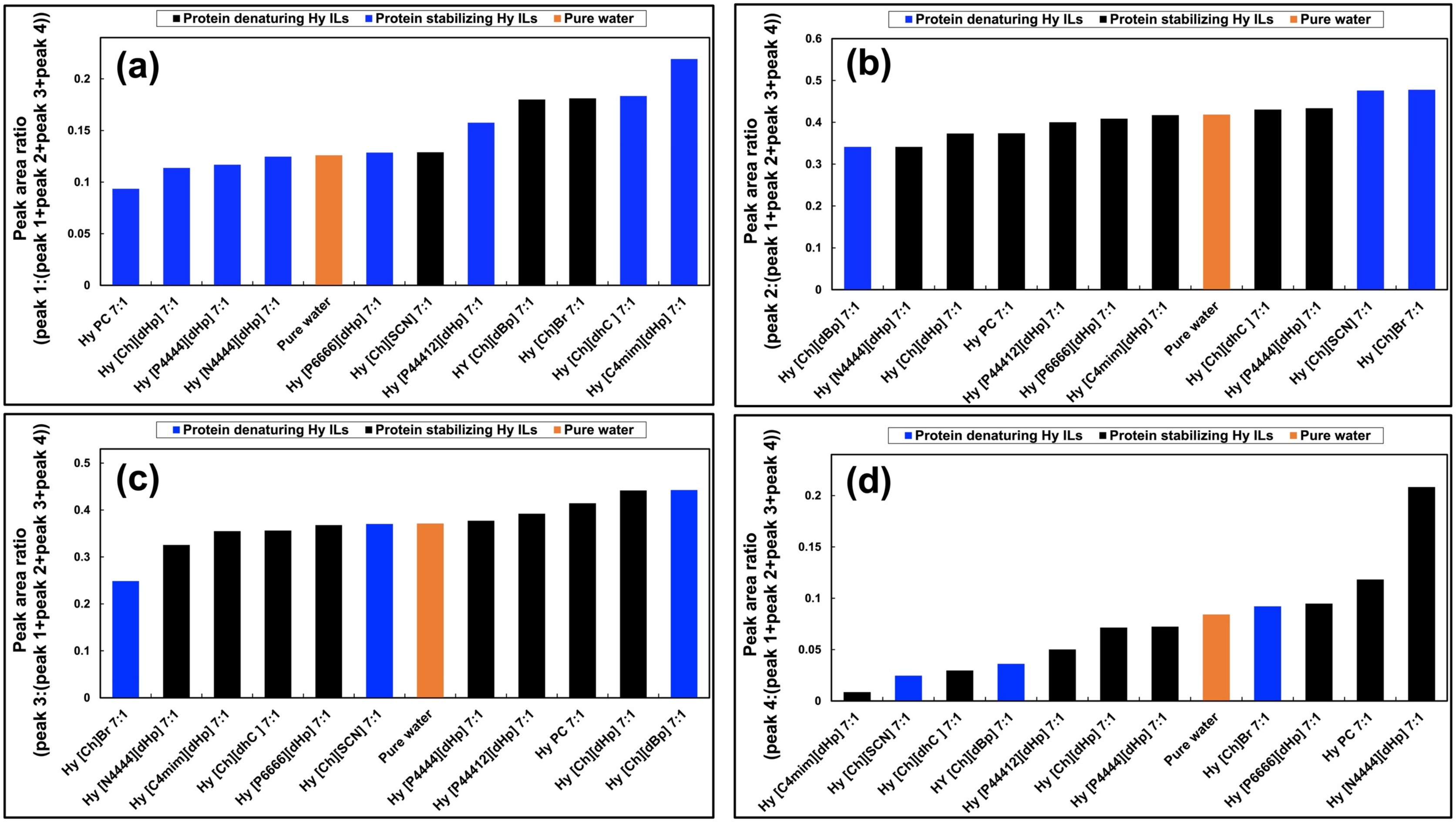

Figure S4. Area ratios of the Gaussian peaks of the OH stretching band of pure water and Hy ILs at a 7:1 molar ratio: (a) area ratio of peak 1 to sum of peaks 1 to 4, (b) area ratio of peak 2 to sum of peaks 1 to 4, (c) area ratio of peak 3 to peaks 1 to 4, (d) area ratio of peak 4 to sum of peaks 1 to 4.

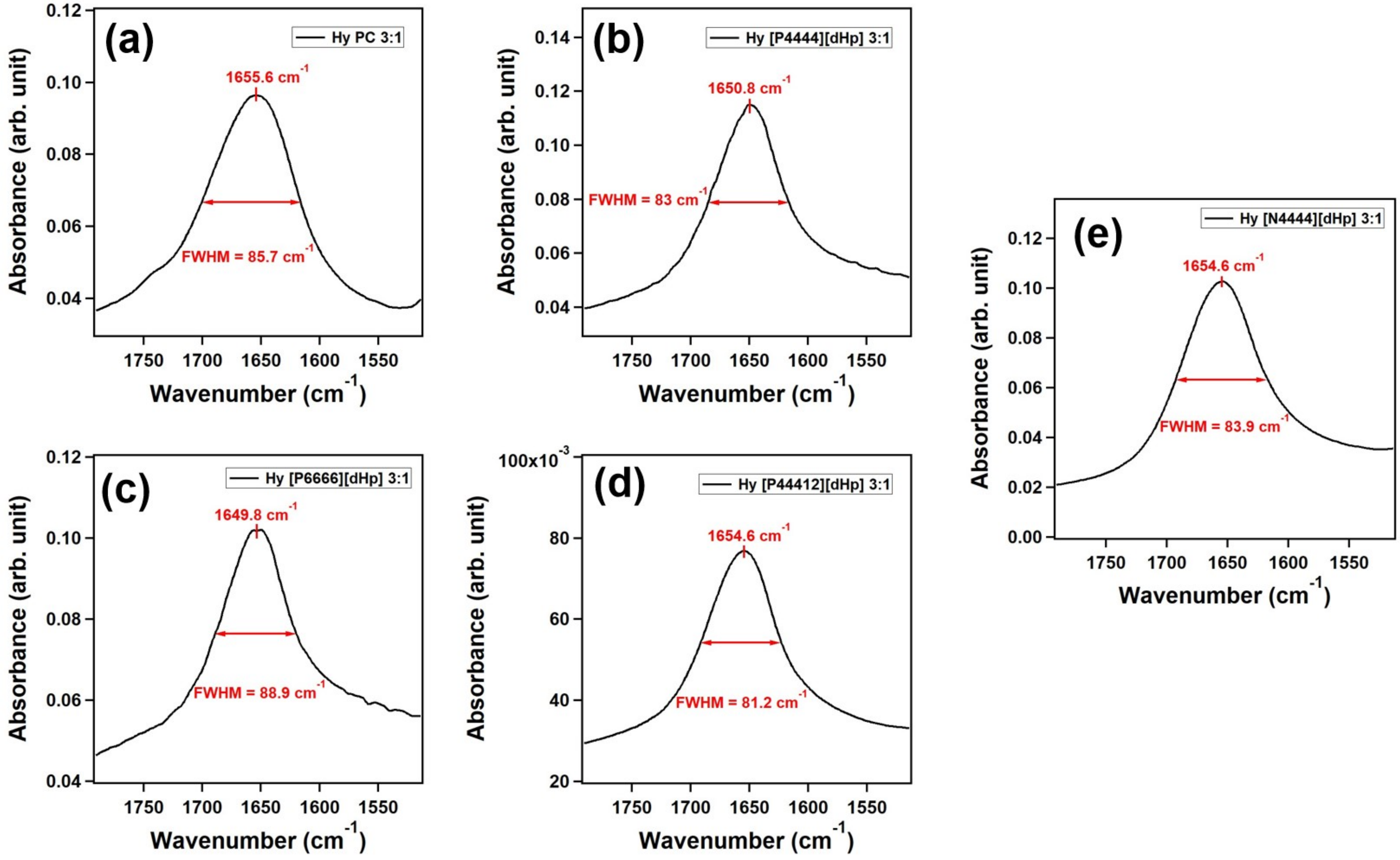

Figure S5. The FWHM of the HOH bending peak of (a) Hy PC, (b) Hy [P4444][dHp], (c) Hy [P6666][dHp], (d) Hy [P44412][dHp], and (e) Hy [N4444][dHp] at 3:1 molar ratio.

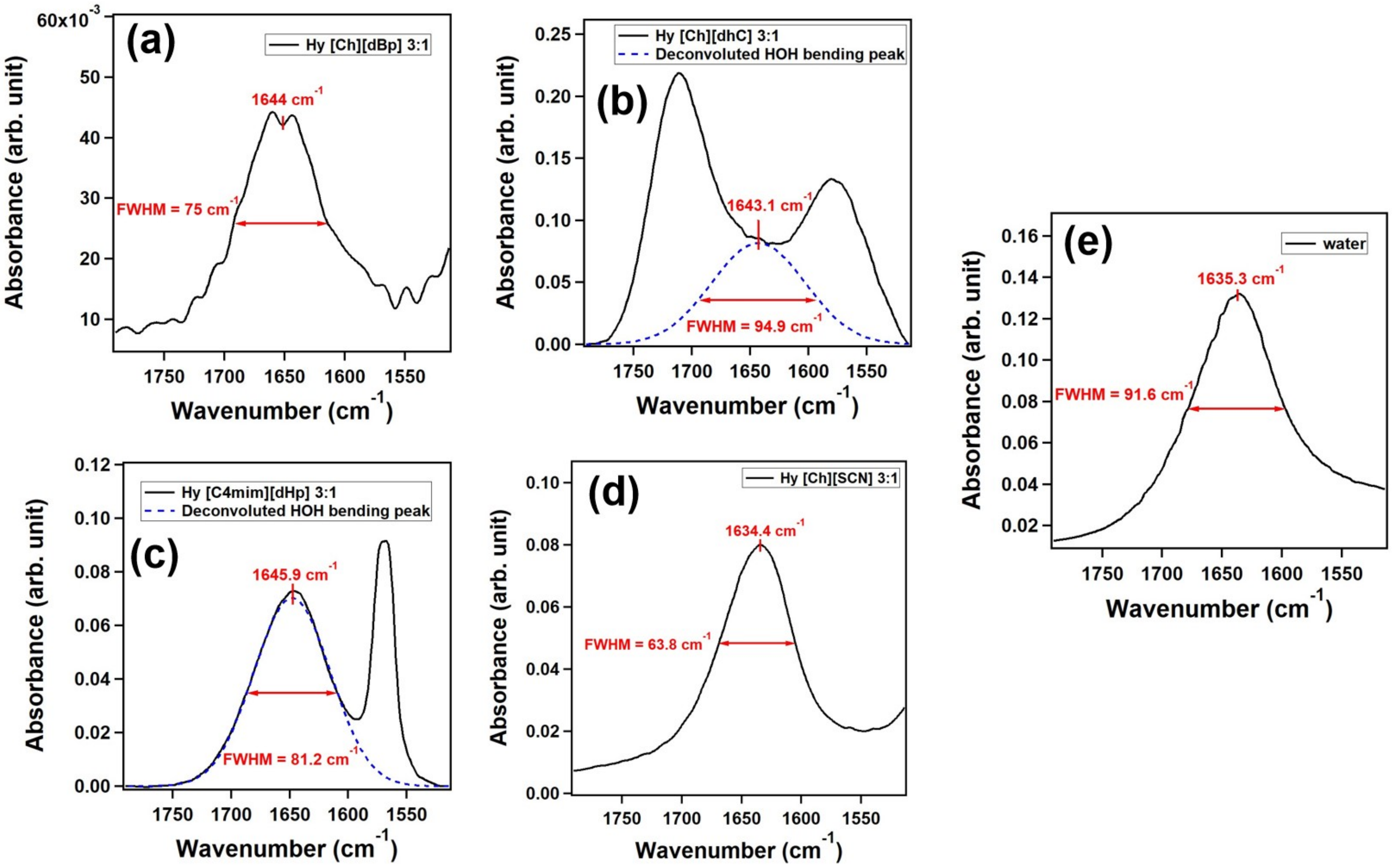

Figure S6. The FWHM of the HOH bending peak of (a) Hy [Ch][dBp], (b) Hy [Ch][dhC], (c) Hy [C4mim][dHp], (d) Hy [Ch][SCN] at 3:1 molar ratio, and (e) pure water. The HOH bending peak position was identified from the second derivative analysis.
